# Identification of QTLs associated with curd architecture in cauliflower

**DOI:** 10.1186/s12870-020-02377-5

**Published:** 2020-04-22

**Authors:** Zhen-Qing Zhao, Xiao-Guang Sheng, Hui-Fang Yu, Jian-Sheng Wang, Yu-Sen Shen, Hong-Hui Gu

**Affiliations:** Institute of Vegetables, Zhejiang Academy of Agricultural Sciences, Hangzhou, 310021 China

**Keywords:** Cauliflower, Curd architecture, Linkage analysis, Genetic map, QTL mapping

## Abstract

**Background:**

Curd architecture is one of the most important characters determining the curd morphology of cauliflower. However, the genetic mechanism dissection of this complex trait at molecular level is lacking. Genes/QTLs responsible for the morphological differences between present-day loose-curd and compact-curd cauliflower haven’t been well revealed.

**Results:**

Herein, by using a common compact-curd parent and two loose-curd parents, we developed two double haploid (DH) populations including 122 and 79 lines, respectively. For each population, we decomposed the curd architecture concept into four parameters (basal diameter, stalk length, stalk angle and curd solidity), and collected corresponding phenotypic data for each parameter across two environments. The Kosambi function and composite interval mapping algorithm were conducted to construct the linkage map and analyze the QTLs associated with curd architecture parameters. A total of 20 QTLs were detected with the minimum likelihood of odd (LOD) values ranging from 2.61 to 8.38 and the percentage of the phenotypic variance explained by each QTL (PVE) varying between 7.69 and 25.10%. Of these, two QTLs controlling stalk length (qSL.C6–1, qSL.C6–2) and two QTLs controlling curd solidity (qCS.C6–1 and qCS.C6–2) were steadily expressed in both environments. Further, qSL.C6–1, qSL.C6–2, qCS.C6–1 and qCS.C6–4 fell into the same chromosomal region of the reference genome, indicating that these loci are involved in pleiotropic effects or are tightly linked.

**Conclusion:**

The current study identified a series of QTLs associated with curd architecture parameters, which might contribute essentially to the formation of present-day loose-curd cauliflower that is widely cultivated in China. These results may pave the way for intensive deciphering the molecular mechanisms of curd development and for marker-assisted selection of curd morphology in cauliflower breeding.

## Background

Cauliflower (*Brassica oleracea var. botrytis* L.), native to coastal regions in Europe, is an important vegetable crop worldwide. As the distinctive commodity organ of cauliflower, curds showed abundant morphological variations, which have been intensively studied at molecular genetic levels [1]. For example, the specific curd phenotype is revealed to be governed by *BoCAL* gene [2, 3], *BoAP1* gene determines the divergence between cauliflower curd and broccoli head phenotypes [4, 5], while the *Or* Gene encoding a plastid associated protein results in an orange curd that is rich in β-carotene [6]. Recently, a genome wide association study based on 174 randomly selected cauliflower gene bank accessions identified a total of 24 significant associations for curd-related traits [7].

One of the most important traits of cauliflower curd is solidity, which is mainly depended on its inside architecture. Compact curds have short stalks, big stalk angles and high solidity, but loose curds show the reverse (Additional file 1: Figure S1). Based on these architecture differences, cauliflower varieties are classified as compact curd and loose curd [8, 9]. Further, the differences of health-promoting compounds and antioxidant capacity between these two curd types were also revealed [10]. The traditional compact-curd cauliflower is cultivated in most regions around the world, while the loose-curd cauliflower is mainly cultivated in China [11]. Loose-curd cauliflower, the main cultivated type in the present China, is characterized by its long and green stem that is rare in the traditional compact type, and also by its distinct edible quality that is popular within Chinese cuisine, such as stir-frying, roasting or hot potting [10]. To our knowledge, the loose-curd cauliflower has been cultivated all over the whole of China, and become an important vegetable species. In addition, this distinct type has been introduced to some other countries of East/Southeast Asia like Vietnam, Thailand and Indian, and has been gradually accepted. Therefore, curd architecture is now of both plant morphology and economic benefits importance [8, 9].

Although a compact curd will become loose from the periphery after maturity, the center of curd is consistently compact, even when the plant becomes bolting and flowering. Varieties always show significant differences in curd architecture. These differences could be already distinguishable when the curd just grows out (Additional file 1: Figure S1). In previous studies, curd solidity has been demonstrated to show significant selection effect and assumed to be governed by number of polygenic factors in an inheritance analysis of the progenies of compact and loose parents [12]. This concept has been partially validated by the genetic background differences revealed between the compact and loose varieties [13]. It has also been postulated that loose-curd cauliflower was derived from the primary cauliflower germplasm that was introduced to China through intense selection on curd characteristics favorable for Chinese cooking habits [13]. However, evidence that artificial selection for curd characteristics, particularly curd architecture, has contributed essentially to the formation of present-day loose-curd cauliflower is lacking. To date, genes/QTLs associated with curd architecture are not well revealed.

Recently, a high-density genetic map including 2741 SNPs has been constructed based on a DH population derived from compact and loose curd cauliflower parents [11]. In the current study, combined with another DH population, we performed the application of this genetic map and population to dissect curd architecture QTLs in cauliflower, and aimed to facilitate a better understanding of curd development.

## Results

### Phenotypic variation of curd architecture related parameters

‘DL3203–61’, the male loose-curd parent of ID population, exhibited bigger basal diameter (mean BD = 19.48 cm), longer stalk (mean SL = 9.00 cm), smaller stalk angle (mean SA = 0.97) and lower curd solidity (mean CS = 1.12) than the female compact parent ‘IL4305’ (mean BD = 16.58 cm, mean SL = 7.39 cm, mean SA = 0.85, mean CS = 2.88) in both environments. Similarly, the male loose-curd parent ‘ZN198’ in IZ population showed mean BD value of 20.92 cm, mean SL value of 9.24 cm, mean SA value of 0.93, mean CS value of 1.75. T-test revealed that parents of compact and loose differed significantly for each the parameter in all experimental environments (Additional file 2: Table S1).

The phenotypic data implied that the four parameters used in the current study could effectively reflect the difference of curd architecture between the loose curd and compact curd. Among the DH lines for each population, largely normal distributions were observed for all parameters. Some DH lines exhibited values outside the corresponding parents, indicating the existence of intergenic interactions.

Both of the two populations showed obvious phenotypic variation of the curd in different planting seasons. The parents and DH lines sowing in August displayed smaller BD and SL values than those sowing in July, whereas the SA and CS values didn’t show clear changes (Additional file 2: Table S1), suggesting that planting season had stronger impact on the curd size rather than the curd texture. The broadsense heritabilities of SL,SA, and CS ranged from 0.72 to 0.98, but for BD, it was calculated only as 0.25 and 0.30 for each population, respectively (Additional file 2: Table S1). Lower heritability of BD heritability indicated that this trait was influenced by environmental factors to a greater degree than the others. Correlation coefficients between different planting seasons for the same parameter in the same population ranged from 0.79 to 0.94 (*P* < 0.001).

### Genetic map construction of ‘ID’ population

**Fig. 1:**
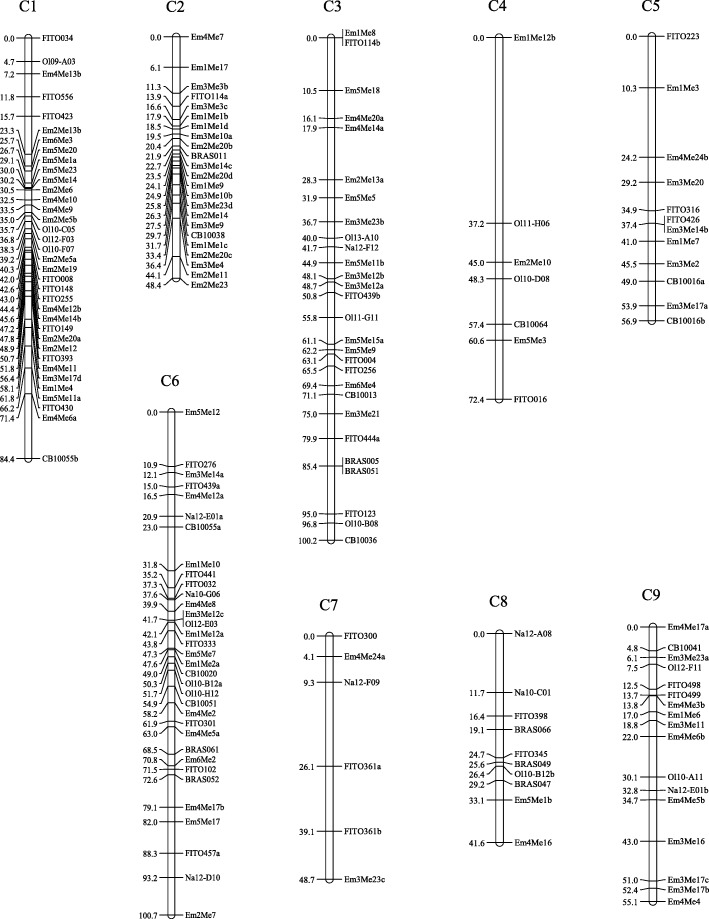
The linkage groups of cauliflower constructed using SSR and SRAP markers. The figures on the left of each linkage group indicate marker positions (cM)

A total of 199 markers exhibited polymorphism between‘IL4305’ and ‘DL3203–61’, showing 8.5% polymorphic ratio between the two parents. One hundred seventy-three markers (77 SSRs and 96 SRAPs) of them were assigned onto 9 linkage groups (LGs) following the linkage analysis (Fig. 1). According to the sequence of SSRs, each LG was anchored to the homologous chromosome of reference genome (http://brassicadb.org/brad/index.php), and was designated the corresponding number (C1-C9). The genetic map spanned a total genetic length of 608.3 cM, with an average marker interval of 3.5 cM. The length of individual LGs varied from 41.6 cM to 100.7 cM, and the marker amounts located on each LG ranged from 6 to 36. This map, hereafter referred to as ‘ID map’, was use to carry out the QTLs scanning for ID population.

Based on the Chi-square test, distorted segregation was observed as 56 of the 173 markers (32.4%) located on linkage map did not accord with the expected 1:1 allelic frequency in ID population (*P* ≤ 0.05). Most of these distorted markers tended to concentrate in specific segment of C2, C8 and C9 linkage groups (Additional file 3: Table S2). In ID population, markers located on C8 and C9 showed higher female parent (IL4305) genotype ratios, while those on C2 displayed higher male parent (DL3203–61) genotype ratios.

### QTL mapping

For ID population, a total of 12 QTLs related to the four parameters were revealed. The LOD values ranged from 2.63 to 8.38 and the PVE varied between 7.69 and 25.10% (Table 1). Among them, one QTL responsible for stalk length (qSL.C6–1) and two QTL related to curd solidity (qCS.C6–1 and qCS.C6–2) could be detected under two environments. Furthermore, the confidence interval qCS.C6–1 and qSL.C6–1 were partly overlapped (Table 1), coinciding with the high correlation between SL and CS. Notably, qSL.C6–1, qSA.C6–1, qCS.C6–1, qCS.C6–1, qCS.C6–1 were identified on the same chromosome 6.

**Table 1:** Putative QTL for curd-traits in ‘ID’ DH population

QTL	Linkage group ^**a**^	Confience interval (cM)	Environment 1 (sowing in July)			Environment 2 (sowing in August)		
			LOD ^**b**^	A ^**c**^	PVE (%)	LOD	A	PVE (%)
qBD.C3–1	C3	21.9–28.3				2.63	1.07	11.35
qBD.C3–2	C3	31.9–38.6				3.06	−1.75	16.68
qSL.C6–1	C6	23.1–35.2	3.51	−0.77	18.95	2.87	−0.41	10.80
qSL.C8	C8	29.2–41.2				2.77	0.51	11.81
qSA.C1–1	C1	0.0–4.7				3.37	−0.06	15.39
qSA.C1–2	C1	21.7–23.3				2.87	0.07	13.03
qSA.C6–1	C6	12.1–25.0	2.73	−0.06	12.40			
qCS.C3	C3	78.9–89.5	4.28	−0.39	14.17			
qCS.C5	C5	22.7–29.5				3.44	−0.30	9.86
qCS.C6–1	C6	18.5–31.8	2.85	0.31	7.69	3.63	0.36	13.04
qCS.C6–2	C6	37.7–47.3	8.38	−0.54	25.10	6.88	−0.46	22.98
qCS.C6–3	C6	54.9–71.6	6.01	−0.40	16.15			

^a^ C followed by a number designates the linkage group where the QTL was detected

^b^ The LOD value detected at peak position

^c^ Additive effect: positive additivity indicate that the QTL allele originated from the parental line ‘IL4305’; negative additivity means that the QTL allele originated from the parental line ‘DL3203-61’

For IZ population, a total of 8 QTLs were identified across two environments. The LOD values ranged from 3.49 to 6.99 and the PVE varied between 11.6 and 25.06% (Table 2). Among them, one QTL governing stalk length (qSL.C6–2) steadily existed in the indicated data of two environments’ trials. The qBD.C1, qSL.C1 and qCS.C1 share almost the same interval on chromosome 1, indicating that these three QTLs might be one locus with pleiotropy. Similarly, qSL.C6–2 and qCS.C6–4 also fell into the same interval on chromosome 6.

**Table 2:** Putative QTL for curd-traits in ‘IZ’ DH population

QTL	Linkage group	Chromosome ID (bp)	Environment 1 (sowing in July)			Environment 2 (sowing in August)		
			LOD	A	PVE (%)	LOD	A	PVE (%)
qBD.C1	C1	34,508,010-37,176,977	4.45	1.22	21.53			
qBD.C5	C5	30,400,023-31,040,767				5.53	−0.82	21.79
qSL.C1	C1	33,237,634-37,176,977	4.48	0.64	22.13			
qSL.C4	C4	14,667,883-18,832,527				3.49	0.30	11.60
qSL.C6–2	C6	30,128,973-34,650,103	6.99	0.56	25.06	6.65	0.61	24.07
qSA.C6–2	C6	7,318,888-9,408,197	4.96	0.02	21.98			
qCS.C1	C1	34,508,010-37,176,977	5.11	−0.65	24.28			
qCS.C6–4	C6	31,839,763-34,650,103				4.50	−0.495	17.11

**Fig. 2:**
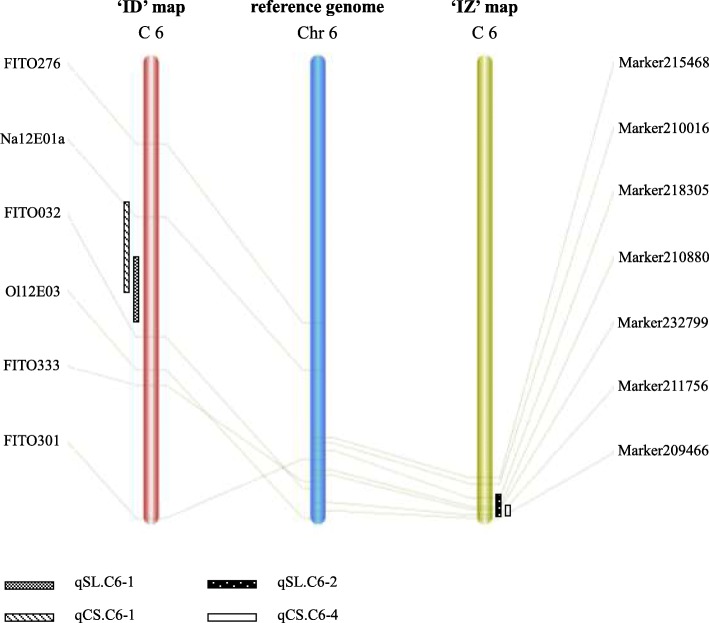
The synteny of the co-localization interval on chrosome C6 among the ‘ID’ map, ‘IZ’ map and reference genome. The length of the bar in the “” symbol with different shading indicates the confidence interval of corresponding QTL

Through comparing between ID map and reference genome, SSR marker FITO276, Na12E01a, FITO032, Ol12E03, FITO333, FITO301 were anchored on chromosome 6. The locus of qSL.C6–2 and qCS.C6–4 were then mapped on the interval of 23,992,639 to 33,263,090 bp of chromosome 6. It is worth to note that the locus of qSL.C6–2 and qCS.C6–4 in IZ map was limited to an interval of 30,128,973-34,650,103 bp of chromosome 6 (Fig. 2). These data suggested that these two loci from different population might point to the same locus/gene on chromosome 6 of *Brassica oleracea* species.

## Discussion

The enlarged curd of cauliflower is considered to be under complex genetic control [14]. Here, we used two DH populations to dissect QTLs responsible for curd architecture. Consistent with previous studies [12, 13], our results demonstrate an obvious heritability of curd architecture, suggesting the feasibility of genetic improvement of stalk length, stalk angle in early generations to adjust the curd morphology.

It is also interesting to note that several QTLs controlling stalk length (qSL.C6–1, qSL.C6–2) and curd solidity (qCS.C6–1, qCS.C6–4) are probably involved in pleiotropic effects. Similar pleiotropic QTLs were widely found in different species [15–18]. In cauliflower, co-localizations of QTLs controlling earliness and curd size were also reported, and were considered to be the result of artificial selection for pyramiding these two agronomic traits from different origins [19]. However, stalk length, stalk angle and curd solidity appears to be the major components responsible for the differences between compact and loose curds, strong correlation among these three parameters has been also revealed in a previous study [20]. The co-localization of QTLs in the current study is therefore more likely to point to the co-domestication of related traits during the loose curd cauliflower domestication and improvement. The two populations used in this study used the same female parent, a compact curd cauliflower inbred line ‘IL4305’, achieved from the compact variety ‘Shengnong 80’ of Fujian province. Between the two different parents, ‘ZN198’, a DH line from the loose curd variety ‘Qingnong 65’, is defined as a ‘typical loose’ curd. ‘DL3203–61’, a DH progeny from the European variety ‘Amazing’, represents another loose-curd type known as ‘semi-loose’ curd. Among the 20 QTLs detected, qSL.C6–1, qCS.C6–1, qSL.C6–2 and qCS.C6–4 were found to locate in the similar region of the reference genome, implying this locus extensively exist in cauliflower from different geographical origin, and govern the curd solidity mainly through regulating the stalk length. From a natural evolutionary standpoint, a loose curd is beneficial for flowering and seed setting for the plants [12]. Nevertheless, artificial selection tend to choice compact curds as the storage and transportation needs. Therefore, the rise of loose curd cauliflower could be regarded as an evolutionary throwback. In any case, the dense distribution of curd architecture QTLs indicates that chromosome 6 of cauliflower genome played an important role in the curd solidity domestication.

Curd development is a pivotal and sensitive stage in the cauliflower lifecycle. The curd architecture is affected to some extent by environmental factors, such as temperature, light and soil fertility [21]. Some previous studies have demonstrated that the curd architecture of certain variety could change more obviously between different planting seasons than that between different planting years [22]. In the current study, we collected phenotypic data of different planting season for each mapping population instead of multiple years’ data, which is commonly regarded as important guarantee for the reliability of QTL detected. The phenotypic data of both DH population showed that the curd size (BD and SL) was generally getting smaller when the sowing date was delayed from July 1 to August 1, whereas the curd shape (SA and CS) didn’t display obvious change trend. This could be explained by the lower temperature and shorter sunshine duration during the curd growth and development period for August 1 sowing than that for July 1 sowing. Across two populations, four QTLs (qSL.C6–1, qSL.C6–2, qCS.C6–1 and qCS.C6–2) could be detected in both planting seasons, indicating their high reliability. In line with the co-localizations of qSL.C6–1, qSL.C6–2 and qCS.C6–1, we assume that the markers located on these regions will provide reliable and powerful tools for marker-assisted selection (MAS) in the improvement of curd morphology, particularly the stalk length, whether the breeding goal is loose curd or compact curd.

Curd architecture is a complex and compound character. It also contains series of other parameters, which could influence the curd morphology and even curd weight. For example, some breeders are trying to select curds with long and thick first-rank stalks as well as thin, short and large number of last-rank stalks, to improve the appearance, yield, resistance to storage and transportation of cauliflower. Therefore, more profound study is desirable to decompose curd architecture concept into more detailed and measurable parameters. The intense genetic research on these factors will provide not only new insights into the molecular mechanisms of curd development, but also powerful tools for the curd improvement in cauliflower breeding programs.

## Conclusions

By using different parameters reflecting curd morphological differences, we demonstrated the curd architecture was inherited in different populations.. Further, a total of 20 QTLs were detected with the LOD values ranging from 2.61 to 8.38 and the PVE varying between 7.69 and 25.10%.An interval showing extensive co-localizations of QTLs responsible for stalk length and curd solidity was revealed on chromosome 6, indicating its important role in the differentiation of present-day loose-curd cauliflower that was widely cultivated in China. These results not only provides novel insights into the diversification of artificial selection leading to different curd architecture (compact/loose) for different demands, but may pave the way for intensive deciphering the molecular mechanisms of curd development. The flanking markers will be valuable for marker-assisted selection (MAS) of curd morphology in cauliflower breeding.

## Methods

### Plant materials and growth conditions

**Fig. 3:**
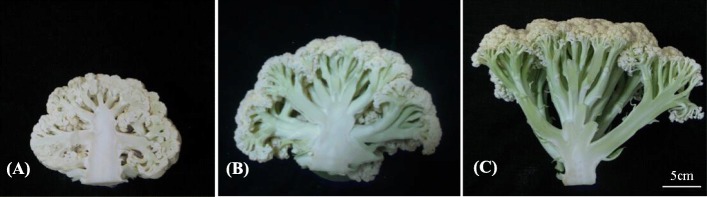
The three parents used in this study. **a**, ‘4305’; **b**, ‘DL3203–61’; **c**, ‘ZN198’

Two DH populations were used in the current study. One including 79 DH lines (‘IZ’ population) developed from the cross ‘IL4305’ × ‘ZN198’ as described by Zhao et al. [11], while the other including 122 DH lines (‘ID’ population) developed by microspore culture from the cross ‘IL4305’ × ‘DL3203–61’. ‘IL4305’ is an advanced inbred line of cauliflower with extreme compact curd generated from a cultivar ‘Shengnong 80’, while ‘ZN198’ and ‘DL3203–61’ are DH lines with different types of loose curd obtained from cultivars ‘Qingnong 65’ and ‘Amazing’, respectively (Fig. 3). Seeds of ‘Shengnong 80’, ‘Qingnong 65’ and ‘Amazing’ were purchased from Wenzhou Shenlu seed Co., Ltd. Field experiments were carried out in two different planting seasons (sowing in July and in August of 2016 for ‘IZ’; sowing in July and in August of 2013 for ‘ID’) in Haining County (HN, 30°320 N, 120°410E). Each experiment had two replicates following the randomized block design. Twelve seedlings per line were planted with 50 cm row spacing and 65 cm line spacing.

### Phenotyping

**Fig. 4:**
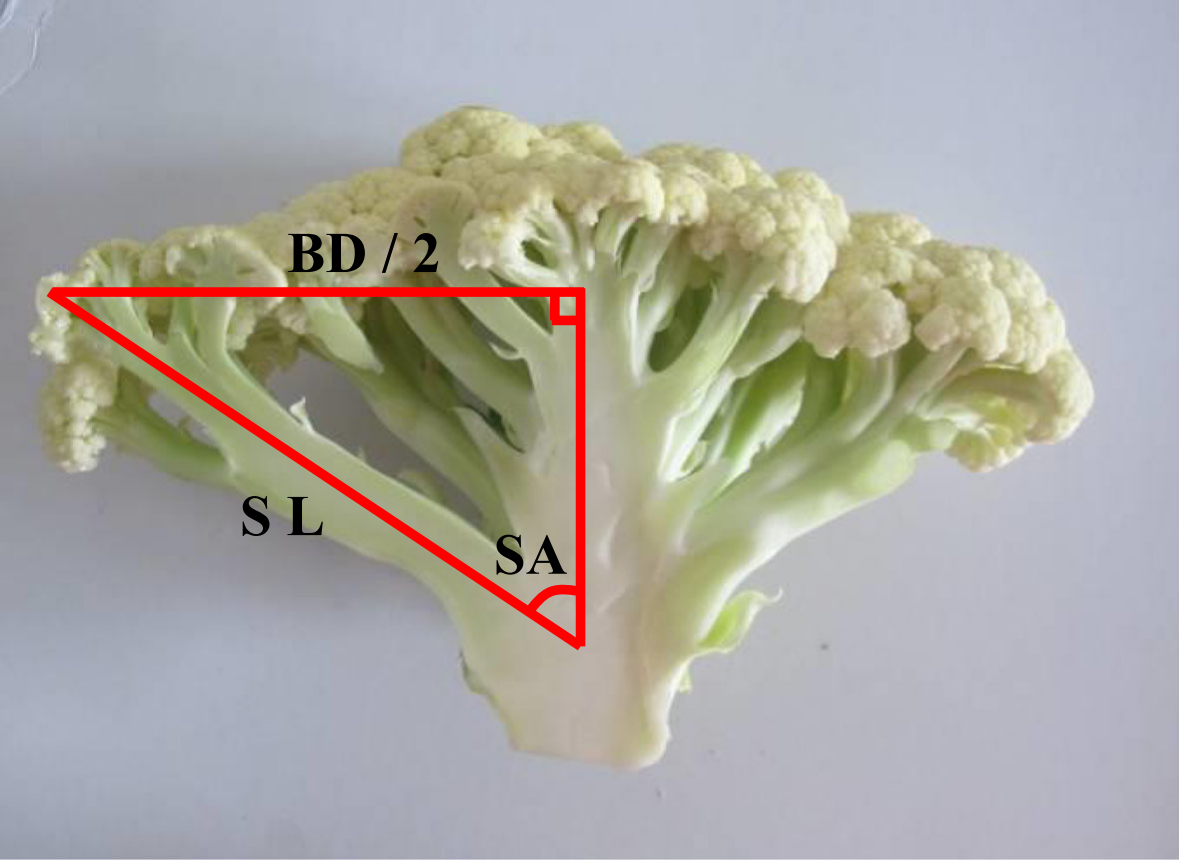
A diagrammatic drawing to illustrate the decomposition of curd architecture

Based on the analysis on phenotype differences between compact and loose curd showed in our previous literature [8], a series of parameters were designed to indicate the curd architecture, including basal diameter (BD), stalk length (SL), stalk angle (SA) and curd solidity (CS). BD was the mean value of the basal diameters measured along three random directions; SL was the average length of four lowest branches, while SA was expressed by the ratio of SL to BD (Fig. 4). CS was assigned by the GR index measured by a texture analyzer TA. TX Plus (Stable Microsystems, England) as described by Zhao et al. [8]. High SA value means small stalk angle, while high CS value represents a solid curd. For each accession, six representative curd samples were harvested and measured 20 days after they were visible, respectively. Generally, a curd will become commercial matured 20 days after it is visible in this planting season. The parameter data of six curds were averaged. Broad-sense heritability for each parameter was analyzed by using SAS v9.3.

### DNA extraction and genotyping

For ID population, young leaves were collected and genomic DNA was extracted from young leaves of 20-days seedling using DNeasy Plant DNA miniprep kits (Qiagen, Hilden, Germany). DNA concentration and quality were detected using an ND-1000 spectrophotometer (NanoDrop, Wilmington, DE, USA) and electrophoresis on 1.0% agarose gel with a standard lambda DNA. A total of 903 SSR primers [23–25] (Additional file 4: Table S3) and 144 pairs of SRAP primer combinations (Additional file 5: Table S4) are used to detect the genotype polymorphism between two parents and then among their progeny DH lines. The PCR reaction was performed following the procedure as described by Zhao et al. [13] for SSR and Mei et al. [26] for SRAP. The amplification fragments were separated in 8% non-denaturing poly-acrylamide gels and detected by using silver staining [27].

For population IZ, the genotype of parents and DH lines were collected based on 2741 SNPs assigned onto the genetic map [11].

### QTL mapping

The Kosambi function of JoinMap 4.0 [28] was used to perform linkage analysis and to build genetic map base on the polymorphic markers in ID population. The LOD and the maximum recombination rate were set as 3.0 and 0.4, respectively.

WinQTLCart 2.5 [29] was used to carry out composite interval mapping for each phenotypic parameter both in ID and IZ population. Drawing function of statistical model was Kosambi, the distance type is Position, and the step size is 2 cM. The thresholds of LOD were determined by 500 permutations at *P* = 0.05. The nomenclature principle of a QTL is to add “q” before the abbreviation of a trait and the linkage group number after the abbreviation. If there are two or more QTLs of one trait on the same linkage group, these QTLs were distinguished by the following Arabic numerals. For example, “qSL.C2–2” indicates the second QTL controlling stalk length on C2 linkage group.

## Supplementary information

**Additional file 1: Figure S1.** The profile of compact curd (left) and loose curd (right) in different curd development stage.

**Additional file 2: Table S1.** Phenotypic performance of each parameter in the DH population.

**Additional file 3: Table S2.** Linkage group regions for allelic frequency skewed from the ratio 1:1 in the DH population.

**Additional file 4: Table S3.** SSR primes used in the ‘ID’ linkage map construction.

**Additional file 5: Table S4.** Sequences of SRAP forward and reverse primers.

## Data Availability

The datasets supporting the conclusions of this article are included in this published article and its supplementary information files. The sequencing data used for QTL analysis in ‘IZ’ map are available in another paper (doi:10.3389/fpls.2016.00334). All the plant materials generated in this study are available from the authors upon reasonable request (Zhenqing Zhao, zhaozq@zaas.ac.cn; Honghui Gu, guhh2199@163.com).

## Abbreviations

DH: double haploid
PVE: Percentage of the phenotypic variance explained by each QTL
LOD: minimum likelihood of odd
