# Supplementary material for: Identification of QTLs associated with curd architecture in cauliflower
Source: BMC Plant Biol. 2020 Apr 22;20:177. doi: 10.1186/s12870-020-02377-5 (PMC7178959; doi:10.1186/s12870-020-02377-5)
Supplement: Supplementary file 1 — Additional file 1: Figure S1. The profile of compact curd (left) and loose curd (right) in different curd development stage. [file 12870_2020_2377_MOESM1_ESM.doc]

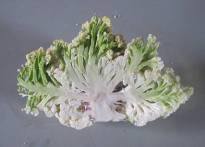

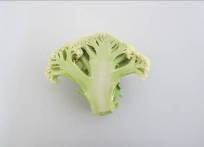

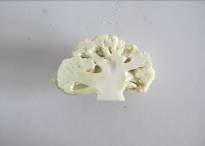

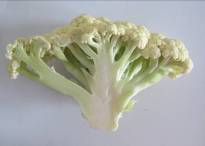

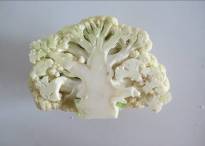

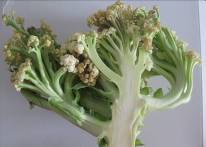


**Figure S1 | The profile of compact curd (left) and loose curd (right) in different curd development stage.**
