# Supplementary material for: Identification of QTLs associated with curd architecture in cauliflower
Source: BMC Plant Biol. 2020 Apr 22;20:177. doi: 10.1186/s12870-020-02377-5 (PMC7178959; doi:10.1186/s12870-020-02377-5)
Supplement: Supplementary file 2 — Additional file 2: Table S1. Phenotypic performance of each parameter in the DH population. [file 12870_2020_2377_MOESM2_ESM.doc]

**Table S1 Phenotypic performance of each parameter in the DH population.**

| Pop. | Parm. | Sowing  date | Female  parent | Male  parent | DH population | | | | Heritability |
| --- | --- | --- | --- | --- | --- | --- | --- | --- | --- |
|  |  |  |  |  | Means | SD | Skew | Kurt |  |
| ID | BD  (cm) | July 1 | 16.88**^a^ | 20.25 | 18.70 | 2.00 | 0.00 | -0.20 | 0.25 |
|  |  | August 1 | 16.28** | 18.70 | 16.78 | 1.55 | -0.30 | 0.71 |  |
|  | SL  (cm) | July 1 | 7.69** | 9.92 | 8.26 | 1.05 | -0.15 | -0.34 | 0.98 |
|  |  | August 1 | 7.08** | 8.08 | 7.31 | 0.86 | 0.35 | 0.62 |  |
|  | SA  (cm/cm) | July 1 | 0.83** | 0.98 | 0.88 | 0.04 | 0.49 | 0.15 | 0.72 |
|  |  | August 1 | 0.86** | 0.96 | 0.87 | 0.06 | 0.87 | 1.81 |  |
|  | CS  (kg/sec) | July 1 | 2.30** | 1.11 | 2.11 | 0.94 | 0.88 | 0.24 | 0.90 |
|  |  | August 1 | 3.46** | 1.13 | 1.97 | 0.88 | 0.40 | 0.51 |  |
| IZ | BD  (cm) | July 1 | 18.83** | 22.63 | 19.67 | 2.88 | 0.44 | 0.32 | 0.30 |
|  |  | August 1 | 16.93** | 19.20 | 17.08 | 1.69 | 0.43 | 1.07 |  |
|  | SL  (cm) | July 1 | 7.83** | 9.78 | 8.47 | 1.34 | -0.26 | 0.47 | 0.96 |
|  |  | August 1 | 7.25** | 8.69 | 7.66 | 0.74 | 0.28 | 1.17 |  |
|  | SA  (cm/cm) | July 1 | 0.83** | 0.95 | 0.82 | 0.07 | 0.43 | 0.06 | 0.88 |
|  |  | August 1 | 0.93* | 0.91 | 0.87 | 0.07 | -0.85 | 0.58 |  |
|  | CS  (kg/sec) | July 1 | 2.48** | 1.76 | 2.11 | 1.02 | 0.86 | 0.16 | 0.78 |
|  |  | August 1 | 2.37** | 1.73 | 1.97 | 0.96 | -0.34 | -0.46 |  |

^a^ The significance level between two parents: *p ≤ 0.05; **p ≤ 0.01.
