# Supplementary material for: Identification of QTLs associated with curd architecture in cauliflower
Source: BMC Plant Biol. 2020 Apr 22;20:177. doi: 10.1186/s12870-020-02377-5 (PMC7178959; doi:10.1186/s12870-020-02377-5)
Supplement: Supplementary file 3 — Additional file 3: Table S2. Linkage group regions for allelic frequency skewed from the ratio 1:1 in the DH population. [file 12870_2020_2377_MOESM3_ESM.doc]

**Table S2 Linkage group regions for allelic frequency skewed from the ratio 1:1 in the DH population.**

| **Linkage group** | **Marker position (cM)** | **Marker number** | **IL4305 (%)** | **DL3203-61 (%)** |
| --- | --- | --- | --- | --- |
| C2 | 16.6-36.4 | 17 | 28.3-37.0 | 61.6-71.2 |
| C8 | 11.7-41.6 | 9 | 71.2-82.2 | 17.8-28.8 |
| C9 | 0.0-22.0 | 10 | 63.0-80.6 | 19.4-36.9 |
