# Supplementary material for: Identification of QTLs associated with curd architecture in cauliflower
Source: BMC Plant Biol. 2020 Apr 22;20:177. doi: 10.1186/s12870-020-02377-5 (PMC7178959; doi:10.1186/s12870-020-02377-5)
Supplement: Supplementary file 4 — Additional file 4: Table S3. SSR primes used in the ‘ID’ linkage map construction. [file 12870_2020_2377_MOESM4_ESM.doc]

**Table S3 SSR primes used in the ‘ID’ linkage map construction.**

| **Prefix** | **Example** | **Primer amount** | **References** |
| --- | --- | --- | --- |
| Na... | Na12-C08 | 196 | Lowe *et al*., 2004 |
| Ol… | Ol10-B03 |
| BRAS... | BRAS055 | 119 | Piquemal *et al*., 2005 |
| CB… | CB10020 |
| FITO... | FITO-036 | 588 | Iniguez-Luy *et al.*, 2008 |
