# Supplementary material for: Identification of QTLs associated with curd architecture in cauliflower
Source: BMC Plant Biol. 2020 Apr 22;20:177. doi: 10.1186/s12870-020-02377-5 (PMC7178959; doi:10.1186/s12870-020-02377-5)
Supplement: Supplementary file 5 — Additional file 5: Table S4. Sequences of SRAP forward and reverse primers. [file 12870_2020_2377_MOESM5_ESM.doc]

**Table S4 Sequences of SRAP forward and reverse primers.**

| **Primers** | **Sequences (5’ - 3’)** | **Primers** | **Sequences (5’ - 3’)** |
| --- | --- | --- | --- |
| Em1 | TGAGTCCAAACCGGATA | Em16 | GAGTATCAACCCGGATT |
| Em2 | TGAGTCCAAACCGGAGC | Em17 | GTACATAGAACCGGAGT |
| Em3 | TGAGTCCAAACCGGATG | Em18 | TACGACGAATCCGGACT |
| Em4 | TGAGTCCAAACCGGACA | Em19 | CACAGTCATGCCGGAAT |
| Em5 | TGAGTCCAAACCGGGAT | Em20 | GACCAGTAAACCGGATG |
| Em6 | TGAGTCCAAACCGGGCT | Em21 | CAGGACTAAACCGGATA |
| Em7 | TGAGTCCAAACCGGTAA | Em22 | ATCAGTCGGACCGGATT |
| Em8 | TGAGTCCAAACCGGTGC | Em23 | GATTGCATCACCGGATG |
| Em9 | TTCAGGGTGGCCGGATG | Em24 | CTTACTTAGACCGGAGT |
| Em10 | TGGGGACAACCCGGCTT | Me1 | GACTGCGTACGAATTATT |
| Em11 | CTGGCGAACTCCGGATG | Me2 | GACTGCGTACGAATTTGC |
| Em12 | GGTGAACGCTCCGGAAG | Me3 | GACTGCGTACGAATTGAC |
| Em13 | AGCGAGCAAGCCGGTGG | Me4 | GACTGCGTACGAATTTGA |
| Em14 | GAGCGTCGAACCGGATG | Me5 | GACTGCGTACGAATTAAC |
| Em15 | CAAATGTGAACCGGATA | Me6 | GACTGCGTACGAATTGCA |
